# Human turn-taking development: A multi-faceted review of turn-taking comprehension and production in the first years of life

**DOI:** 10.3758/s13423-025-02749-8

**Published:** 2025-08-07

**Authors:** Samuel H. Cosper, Simone Pika

**Affiliations:** 1 Chair of Lifespan Developmental Neuroscience, Faculty of Psychology, Technische Universität Dresden, Dresden, Germany; 2 Comparative BioCognition, Institute of Cognitive Science, Osnabrück University, Osnabrück, Germany; 3 Center for Early Childhood Development and Education Research (CEDER), Osnabrück University, Osnabrück, Germany

**Keywords:** Social interactions, Conversational turn-taking, Developmental milestones, Phylogenetic perspective

## Abstract

Human communication builds on a highly cooperative and interactional infrastructure—conversational turn-taking. Turn-taking is characterized by reciprocal, alternating exchanges between two or more interactants, avoidance of overlap, and relatively short response times. Although the behavioral principles governing turn-taking in spoken interactions of human adults have been investigated for decades, relatively little is known about the acquisition of conversational turn-taking skills and the developmental trajectories of turn-taking comprehension and production. The aim of the present review was to provide a comprehensive overview of turn-taking development enabling the extrapolation of developmental milestones and investigations across species and taxa. it thus aims to serve as a crucial guide to our current understanding of turn-taking in childhood and instigate a better understanding of turn-taking phylogeny, its evolutionary roots, as well as systematic, quantitative applications across and between species, thereby possibly bridging the existing gap between linguistic and nonlinguistic species.

## Introduction

Human life is permeated by cooperative social interactions that are important for adult intercommunication and societal rituals, as well as for early language development (Çetinçelik et al., 2021; Levinson, 2006, 2016; Ratner & Bruner, 1978). These interactions are characterized by the perception of roles and expectations, and involve the exchange of multimodal information, such as visual, tactile, and auditory information (Levinson, 2006). Cooperative social interactions typically involve two or more interactants and are largely regulated by specific roles for those involved, be it, for example, a speaker and a hearer or an actor and a spectator (Sacks et al., 1974). Participants take *turns* in a manner of exchanging these interactional roles with *turn-taking* ranging from conversations, to playing games, learning environments, cultural ceremonies, sports, and even behaviors at traffic intersections (Sacks et al., 1974; Skantze, 2021). Sacks et al. (1974) were the first to propose a systematics of the organization of conversational turn-taking pinpointing *fundamental features* such as the use of specific time windows between turns, avoidance of overlaps, and utilization of adjacency pairs (Schegloff, 2007). Subsequently, turn-taking behavior, specifically in question–response sequences, has been studied across several cultures and languages, suggesting universal patterns in the underlying structure of response latency in conversation (Stivers et al., 2009). A more recent reinvestigation of the pattern of timing between turns including 24 languages and a higher number of items in the speech corpora yielded less variation between languages and cultures, with a mean transitional time of 59 ms (Dingemanse & Liesenfeld, 2022). Furthermore, recent research reports on fundamental concepts, cues and elements involved in conversational turn-taking (e.g., Levinson, 2016; Skantze, 2021) and their development in infancy and childhood (e.g., Bakker et al., 2011; Casillas & Frank, 2017; Donnelly & Kidd, 2021; Hilbrink et al., 2015; Kalashnikova & Kember, 2020; Keitel & Daum, 2015; Keitel et al., 2013; Smith & McMurray, 2018; Thorgrimsson et al., 2014, 2015). However, a comprehensive, concise overview of turn-taking development and involved milestones is currently nonexistent, hampering an in-depth understanding of the development of this crucial mechanism, its involved elements, and informing comparative studies tackling its phylogeny.

Hence, the main goal of the present review paper was to compile the most crucial findings from the existing body of research on turn-taking development, to pinpoint developmental milestones, and to link these findings to enable a better understanding of the evolutionary trajectory of turn-taking based on a recent comparative framework (cf. Pika et al., 2018). As there have been a considerable number of publications on conversational turn-taking—and we can only cite some of them—we have restricted our selection of citations to publications that either cite secondary literature or are exemplary for general phenomena. In order to identify such publications, we searched the electronic databases Web of Science and Google Scholar with terms including “child” OR “children” OR “toddler*” OR “infan*” AND “turn-taking” OR “turn taking” OR “conversational turn-taking” OR “conversation” OR “gap” OR “overlap” OR “timing” OR “gesture” OR “gaze” AND “comprehension” OR “production.”

We restricted the searches to publications in English, and primary screening was done based on the title and abstract. More detailed screening was conducted by reading all publications. We only included those publications which yielded novel contributions, while also citing any reviews found on each subtopic. We considered novel contributions to be any article that presented new data, a new analysis, corrections to previous publications, new questions to previous data, or replications in which findings differed from previous reports. As such, replications of previous reports that only confirmed previous findings were not included. Additional forward and backward citation searches were performed to include any publications not identified in the electronic searches.

In this review, we will first briefly dive into the history of conversational turn-taking, provide information about the most predominant and promising methodological approaches used thus far, and pinpoint current limitations and research biases. Subsequently, we will present an overview of the existing research on turn-taking development with a special focus on turn-taking comprehension, turn-taking production, and developmental milestones. Subsequently, we will introduce and link the existing findings to a recent comparative framework, enabling direct, systematic comparisons between species and taxa and thus an informed understanding of the phylogenetic history of turn-taking.

### A brief history of research into conversational turn-taking

Turn-taking has predominantly been studied via conversation analysis (CA), which is a primarily qualitative form of micro-analysis of human social action originating from various research strands within the social sciences (Garfinkel, 1967; Goffman, 1964). During the past 2 decades, however, research on turn-taking interactions of human adults and children and underlying cognitive skills has stirred research attention in other scientific fields including sociology, linguistics, cognitive science, neuropsychology, and animal behavior (Holler et al., 2015; Levinson, 2016; Logue & Stivers, 2012; Rohlfing et al., 2020; Rossano, 2013; Skantze, 2021).

The first seminal article on turn-taking and implications for conversation dates back to the last century and was written by two sociologists and a linguist (Sacks et al., 1974). It proposed an economic set of guidelines that govern the alternating nature of conversation between two or more individuals. First, the authors identified a set of 14 facts they deemed necessary for any model of conversational turn-taking (hereafter, turn-taking) to be considered of merit (see Table 1). These consisted of information and constraints on when turns occur, how turns are organized between conversational partners, how conversational partners know when to take a turn, how long turns can be, the timing between turns, and what happens when turn-taking errors occur (Sacks et al., 1974). These facts were subsequently used to construct a system of turn-taking composed of two components and a distinct set of rules, either accommodating the facts directly or allowing for a derivation thereof (Sacks et al., 1974). The first component, turn-constructional component, indicates that turns are comprised of linguistic unit-type constructions of all syntactic levels—sentence, clause, phrase, word—and that the speaker’s completion of any of the units in their proper context can be categorized as a possible transition-relevance place, allowing for turn-taking to occur. The second component, the turn-allocational component, describes how the next turn is delegated—either by means of the current speaker or by self-selection. The rules then describe how turn allocation should occur: instances of only current speaker selecting the next speaker, when both the current speaker can select and a self-selection can occur, and when the current speaker cannot select the next speaker and only either a self-selection or a continuation may occur. The second rule identifies what happens when a continuation occurs and then when again speaker selection is appropriate.

**Table 1:** A list of facts necessary for a model of turn-taking from Sacks and colleagues (1974, pp. 10–11)

Number	Description
Fact 1	Speaker change recurs, or at least, occurs
Fact 2	Overwhelmingly, one party talks at a time
Fact 3	Occurrences of more than one speaker at a time are common, but brief
Fact 4	Transitions from one turn to a next with no gap and no overlap between them are common. Together with transitions characterized by slight gap or slight overlap, they make up the vast majority of transitions
Fact 5	Turn order is not fixed, but varies
Fact 6	Turn size is not fixed, but varies
Fact 7	Length of conversation is not fixed, specified in advance
Fact 8	What parties say is not fixes, specified in advance
Fact 9	Relative distribution of turns is not fixed, specified in advance
Fact 10	Number of parties can change
Fact 11	Talk can be continuous or discontinuous
Fact 12	Turn-allocation techniques are obviously used. A current speaker may select a next speaker (as when a current speaker addresses a question to another party); parties may self-select, in starting to talk
Fact 13	Various “turn-constructional units” are employed. Turns can be projectedly “one work long,” or, for example, they can be sentential in length
Fact 14	Repair mechanisms for dealing with turn-taking errors and violations obviously are available for use. For example, if two parties find themselves talking at the same time, one of them will stop prematurely, this repairing the trouble

This turn-taking model (Sacks et al., 1974) has since been applied and tested in CA, as well as expanded into the fields of linguistics, social psychology, and developmental psychology (cf. Holler et al., 2015; Levinson, 2016; Rohlfing et al., 2020; Skantze, 2021). In respect to the alternating nature of human communication, conversations are characterized by *turns*, where two or more participants alternate speaking. While conversational turns have been described to last about two seconds (cf. Levinson, 2016; Levinson & Torreira, 2015), the length of a turn can drastically vary and the flexibility of turn length is fundamental to turn-taking and communication (Levinson, 2016; Levinson & Torreira, 2015; Sacks et al., 1974). When collecting research findings on what is currently known behaviorally and experimentally about the turn-taking system, Levinson and Torreira (2015) showed in their review paper that while longer gaps and overlaps do occur, the timing of turn-taking in human conversation does indeed match the model outlined by Sacks and colleagues (1974). The typical time passed between speakers, also known as an *interspeaker* gap (cf. Levinson & Torreira, 2015; Stivers et al., 2009), is around 200 ms with variation. A recent investigation on 24 languages, however, suggested that this time can even be much faster in adult speech interactions with an average length of 59 ms between turns (Dingemanse & Liesenfeld, 2022). These relatively short temporal gaps between turns are surprising, as it takes approximately 600 ms to encode a spoken word (Indefrey & Levelt, 2004; Levinson & Torreira, 2015). This means that not only do hearers have to process what the speaker is saying but they also simultaneously plan their speech response within a conversation to transition between speakers with little gap or overlap. Hence, interactants as well as third-party spectators may rely on and use various cues to assess when turn-taking is going to occur.

Recently, Skantze (2021) provided an overview on the types of cues that occur during turn-taking in adult interactants, how and when they are implemented, and what effect they may have on turn-taking. He showed that both speakers and hearers in a conversation are aware of turn-taking cues that indicate when a turn is going to end—*turn-yielding cues*—and when a turn is being held—*turn-holding cues* (Duncan, 1972; Duncan & Fiske, 1977; Duncan & Niederehe, 1974). These cues are useful markers for turn-taking, fostering minimal overlap and minimal temporal gaps.

Skantze (2021) distinguished between.

- *verbal cues*, including syntactic, semantic, pragmatic information, as well as fillers for filled pauses;
- *prosodic cues*, including intonation and vocal quality;
- *breathing cues,* including inhalation and exhalation;
- *gaze,* including toward the current speaker, away from the current speaker, and to the next speaker; and
- *gestures*, including manual gestures.

Turn-taking can also be considered from a cross-cultural perspective. A seminal investigation into gap length between utterances was comprised by Stivers and colleagues (2009), investigating cultural-based language differences in the realization of timing in turn-taking. By comparing yes–no questions, ranging from 95 to 247 questions per language in 10 different languages, the authors showed that while turn-taking may be realized in certain language communities slightly differently, there is a general universal for gap length that emerges and the mean response time between turns is at about 200 ms across languages, with language-specific means falling within 250 ms of the overall mean. Recently, Dingemanse and Liesenfeld (2022) expanded upon this study by investigating a language corpora including 24 languages of at least 1,000 turns per language. In contrast to Stivers and colleagues (2009), they found a much lower variation with mean response latencies of 59 ms, with 46% of the investigated languages showing a slight overlap. A further analysis, more directly comparable to Stivers and colleagues (2009), including data of 10 languages with at least 250 question–answer sequences did not radically differ from the mean response time of the overall analysis (Dingemanse & Liesenfeld, 2022). Such discrepancies can be interpreted as not only the mean temporal latencies themselves being important but also to what leniency specific cultures consider utterances as being temporally contingent. This, however, is only an interpretation and would need to be validated in future studies with more languages included. Levinson (2006) also highlighted the importance of culture and interaction, indicating how interaction at the sociocultural level may “play a central role in higher level social processes” (Levinson, 2006, p. 62), including social institutions and international politics.

Finally, to contextualize the involved cognitive complexity, and cross-cultural similarities and differences of turn-taking, it is crucial to gain insights into its phylogenetic history. For instance, Levinson (2016) postulated that cooperative turn-taking plays an integral role in the “Universal Infrastructure for Language,” (Levinson, 2016, p. 6) with the layered system of language building on preexisting mechanisms and cognitive skill. Pika and colleagues (2018) expanded upon this concept by providing a detailed review on turn-taking abilities across nonhuman species and developing a comparative framework of turn-taking to tackle similarities and differences between human and nonhuman turn-taking interactions. Abreu and Pika (2022) further expanded on this, providing an overview of turn-taking development in nonhuman mammal species, while Verga and colleagues (2023) carried out an overview of the evolution of timing in social interaction, including a theoretical and empirical framework with species-appropriate paradigms.

### Methodological approaches

Several methods have been used to investigate turn-taking in both human adults and children during development. In this section, we will provide a brief overview of established methods used across decades as well as more recent methods (e.g., eye-tracking: Augusti et al., 2010; vocal recordings: Bloom et al., 1987; video recordings of interactions: Craig & Gallagher, 1982; observation-based gaze recordings: Crown et al., 2002; LENA® vocal recordings: Donnelly & Kidd, 2021; electroencephalography: Lam-Cassettari et al., 2021; video recordings and sensor mats: Reddy et al., 2013). The latter especially provide an understanding of turn-taking with regard not only to the production but also the comprehension of turn-taking, thereby enabling insights into the underlying cognitive and neuropsychological mechanisms of turn-taking (cf. Lam-Cassettari et al., 2021; Liao et al., 2015). The addition of neuropsychological mechanisms is of particular importance, as many social-communicative behaviors have identified neural correlates (for a review, see Cruz et al., 2020). Historically, and also carrying on today, many studies have been conducted utilizing observational situations (e.g., Beebe et al., 1988; Bloom et al., 1987; Gratier & Devouche, 2011; Henning et al., 2005; Hilbrink et al., 2015; Rosenthal, 1982; Rutter & Durkin, 1987). Here, experimenters have studied turn-taking interactions during conversations, predominantly focusing only on involved spoken signals by video or audio recordings and in some cases the transcriptions of the interactions (e.g., Bedrosian et al., 1988; Beebe et al., 1988; Bloom et al., 1987; Craig & Gallagher, 1982; Dominguez et al., 2016; Gallagher & Craig, 1982; Gratier & Devouche, 2011; Gratier et al., 2015; Henning et al., 2005; Hilbrink et al., 2015; Rosenthal, 1982; Rutter & Durkin, 1987; Snow, 1977). Observational studies varied from individuals engaging in specific tasks (e.g., Goldstein et al., 2009; Nomikou et al., 2017; Rochat et al., 1999; Rocissano et al., 1987) to free-conversation situations (e.g., Bedrosian et al., 1988; Beebe et al., 1988; Bloom et al., 1987; Craig & Gallagher, 1982; Dominguez et al., 2016; Gallagher & Craig, 1982; Hilbrink et al., 2015).

Related to observational studies is the method of corpus analysis that use expansive corpora of transcribed conversations, largely acquired through observational studies, with which researchers are able to gather and analyze large samples of turn-taking in natural environments (e.g., Casillas et al., 2016; Donnelly & Kidd, 2021). Donnelly and Kidd (2021) created their own database using the Language Environment Analysis (LENA®) technology, allowing for the recording and analysis of large data—up to 16 h per recording (cf. Greenwood et al., 2011). Such approaches allow for generalizations to be made about turn-taking, particularly pertaining to validations of productive models of turn-taking, for example, the model by Sacks and colleagues (1974), as well as for the investigation of underlying mechanisms across cultures and languages. Similarly, corpora analysis not only is used for the cultivation of large datasets through uniform measures of turn-taking but can also be used to investigate the underlying cognitive mechanisms of turn-taking or broader mechanisms behind conversation production and comprehension by means of casual inference tools or cognitive modelling. While big data and large language models (LLMs) cannot explain all phenomena of turn-taking and also have their limitations, they are very useful for the investigation of many claims and for the further understanding of general aspects of turn-taking, which may not be able to be robustly measured in an experimental design.

Turn-taking skills have also been explored experimentally. For instance, researchers manipulated specific aspects of turn-taking, such as cues and timing (i.e., gaps and overlaps), to understand when turn-taking is used and how it is perceived (e.g., Bakker et al., 2011; Casillas & Frank, 2017; Clark & Lindsey, 2015; Kalashnikova & Kember, 2020; Keitel & Daum, 2015; Lam-Cassettari et al., 2021; Lammertink et al., 2015; Liao et al., 2015; Nagy & Molnar, 2004; Reddy et al., 2013; Thorgrimsson et al., 2014, 2015; von Hofsten et al., 2009). Furthermore, recently advanced methodologies such as eye-tracking devices, and electroencephalography (EEG) have been applied. Eye-tracking studies have for instance been used to investigate cognitive effects of attention, the facilitation of learning, task processing and engagement, cognitive control, decision-making, and familiarity versus novelty effects (Augusti et al., 2010; Bakker et al., 2011; Casillas & Frank, 2017; Çetinçelik et al., 2021; Eckstein et al., 2017; Kalashnikova & Kember, 2020; Keitel & Daum, 2015; Keitel et al., 2013; Lammertink et al., 2015; Sekicki & Staudte, 2018; Thorgrimsson et al., 2014, 2015). To this end, the use of eye-tracking technology has provided insights into the predictive elements of turn-taking, such as gaze shifting to the speaker, before the turn has been actively taken (cf. Rutter & Durkin, 1987). EEG, in contrast, has been used to better understand the cognitive and neurophysiological mechanisms of turn-taking, such as the cognitive demands of simultaneous listening and utterance planning as they pertain to specific turn-yielding cues—for example, semantic and pragmatic indicators (Bögels, 2020; Bögels et al., 2018; Lam-Cassettari et al., 2021; Liao et al., 2015). Similar to observational studies, corpora analysis and LLMs can be used to investigate specific questions as well as broader interactions between different turn-taking cues. Liesenfeld and Dingemanse (2022) provided an overview on how to build and curate conversational corpora, and they also gave insights on how empirical questions can be answered with language-specific and cross-cultural corpora without the need to collect new data. Further examples of empirical questions that can be asked are, for instance, 1) how frequent are specific cues in turn-taking and age and culture/language differences, 2) how do various turn-taking cues dynamically interact with one another both during development and in adulthood, 3) how turn-taking repairs are enacted and frequency of repair mechanisms, 4) turn-taking in human–robot interactions, and 5) gestural and multimodal responses in turn-taking (cf. Dingemanse & Liesenfeld, 2022; Liesenfeld & Dingemanse, 2022; Rohlfing et al., 2020; Skantze, 2021). Each of the methodologies are further presented in the “Turn-Taking Comprehension” and “Turn-Taking Production” sections, below, concerning the comprehension and production of turn-taking.

### General development of turn-taking in childhood

Like the development of prosody, phonology, semantics, and syntax (e.g., Friedmann & Rusou, 2015; Iverson, 2010; Ruben, 1997; Werker & Hensch, 2015; West & Iverson, 2021), the development of turn-taking skills takes place over the course of childhood (Ervin-Tripp, 1979; Hilbrink et al., 2015) and has been suggested to reach adult-like maturity around the age of 6 years (Ervin-Tripp, 1979). It is important to note that the claim that turn-taking development stagnates at 6 years of age has not been corroborated by any further publication. Although the majority of language learning and the development of involved communicative processes takes place after birth, language acquisition in the form of language processing already starts in the fetal brain whilst in utero. For instance, studies investigated speech preferences at birth and showed that neonates have a preference for their mother’s voice (DeCasper & Fifer, 1980; DeCasper & Spence, 1986; Panneton & DeCasper, 1986) as well as for the prosodic structure of their native language (Demany, 1982; Demany et al., 1977). While the preference of auditory stimuli and prosodic features of the native language are vital for the development of language, other aspects of human development must also be considered to investigate the emergence and development of the inherent social aspects of turn-taking. For instance, human interaction may be dominated by speech, yet speech does not code every action within a given conversation (cf. Levinson, 2006). This phenomenon leaves many actions to be inferred or indirectly communicated by means of facial expressions, gestures, postures, speech acts (including locutionary, illocutionary, and perlocutionary acts; Austin, 1975; Searle, 1979), and the non-language-related mechanisms of perceiving these actions and signals (Levinson, 1983, 2006; Sperber & Wilson, 1986). Furthermore, some scholars suggested that elements characterizing turn-taking interactions may have different evolutionary and ontogenetic origins, with some possibly occurring earlier respectively later during development (Abreu & Pika, 2022; Levinson, 2006; Pika et al., 2018; Sacks et al., 1974).

In 2006, Levinson postulated the *interaction engine* hypothesis, which proposed that the structure of human social interaction forms a cognitive-and-ethological foundation of many cognitive abilities and behavioral dispositions that belong to human communication. Properties involved include interactions, responses to actions or intentions, cooperation, action sequences, participation structures, temporal expectations of reciprocated actions, and turn-taking across modalities, all developing even before language is fully acquired (Levinson, 2006). Levinson (2016) further proposed that turn-taking precedes language in ontogeny and possibly also in phylogeny. Thus, despite differences in the production and comprehension of turn-taking in childhood and adulthood (Hilbrink et al., 2015), young infants are growing up in a world of frequent social interactions and have some features of turn-taking at their disposal already as neonates (e.g., temporal relationships, avoidance of overlap). However, although the interaction engine stresses the acquisition of turn-taking skills in early social settings, another important area for future research is to gain a general understanding of the development of turn-taking cues, both concerning their comprehension and their production. As such, in the current review, we categorize turn-taking abilities by means of general social interaction, comprehensive turn-taking, and productive turn-taking to be able to have an informed overview of the development of turn-taking and focus on a single aspect of turn-taking at a time.

### Limitations and research biases

Although a considerable body of research on the development of turn-taking exists, there are distinct limitations hampering an in-depth understanding of turn-taking development. For instance, 1) there is a clear lack of longitudinal studies, 2) the role of gestures and specifically multimodal signals has been widely ignored (both as input and as responses), 3) possible correlations between development and types of turn-taking cues have not been investigated (e.g., a negative, inhibitory correlation or a positive, facilitating correlation), 4) cultural differences in the development of turn-taking have rarely been investigated, and 5) there is a lack of a culturally sensitive and standardized coding scheme and a lack of cohesion across experimental designs.

For instance, concerning the first limitation—lack of longitudinal studies—some studies have systematically examined development over the course of the first months and years of life (Fusaroli et al., 2024; Hilbrink et al., 2015; Lindsay et al., 2019; Nomikou et al., 2017; Rochat et al., 1999; Rome-Flanders & Cronk, 1995; Rutter & Durkin, 1987; Smith & McMurray, 2018). While this list (see Table 2) only consists of the studies included in this review and is therefore not exhaustive, it emphasized the lack of longitudinal developmental studies and the research bias on specific age groups. Furthermore, the presented longitudinal studies focused on a specific aspect of turn-taking only (e.g., timing of utterances, gaps, gaze), while virtually nothing is known about how the developments of these specific aspects affect or correlate to other aspects of turn-taking. While the inclusion of all aspects in a singular paper may not be feasible, a consortium such as the ManyBabies Consortium (cf. Frank et al., 2020) or open science projects may be able to integrate far more aspects of turn-taking and create robust, generalizable, and detailed analyses and results across cultures and age groups, solidifying our understanding of the development and nature of turn-taking. Furthermore, not all of the above longitudinal studies focus on similar or comparable age groups, leaving gaps in the literature in regard to longitudinal development under a single paradigm. The inclusion of longitudinal studies would allow for individual development to be assessed, allowing for not only generalizations, but also to better understand statistical deviations between individuals, groups, and even cultures. This, however, should be done with a proper cross-cultural design (cf. Wen et al., 2025).

**Table 2:** A list of longitudinal studies (included in the current review) investigating turn-taking along with the age groups included within each study (TD = typically developing and ASD = autism spectrum disorder)

Author/Year	Ages included in longitudinal study
Fusaroli et al. (2024) Hilbrink et al. (2015)	TD: 20-, 24-, 28-, 32-, 36-, and 40-month-olds ASD: 32-, 36-, 40-, 44-, 48-, 52-month-olds 3-, 4-, 5-, 9-, 12-, and 18-month-olds
Lindsay et al. (2019)	36–60-month-olds
Nomikou et al. (2017)	4- and 6-month-olds
Rochat et al. (1999)	2-, 4-, and 6-month-olds:
Rome-Flanders and Cronk (1995)	6-, 9-, 12-, 15-, 18-, and 24-month-olds
Rutter and Durkin (1987)	12-, 18- and 36-month olds and 9–36-month olds
Smith and McMurray (2018)	4–60-month-olds

Concerning the second limitation—the role of gestures and multimodal signals—the existing studies on turn-taking development mainly focused on the single communicative modality of speech only (cf. Levinson, 2024; Rohlfing et al., 2020; Skantze, 2021), thereby ignoring that language is an integrated system of gesture and speech (cf. Levinson, 2006; McNeill, 1992). Recently, however, some scholars started to investigate turn-taking development in the gestural modality and provided evidence for the importance of gesture in the production and comprehension of turn-taking at a young age (e.g., Clark & Lindsey, 2015; Thorgrimsson et al., 2014, 2015). Specific coding schemes have been introduced to allow for objective coding of multimodal signals in joint attention, which may serve as a guide for turn-taking (Gabouer & Bortfeld, 2021; Gabouer et al., 2020). The integration of specific coding schemes for turn-taking would not only foster the consideration of multimodal cues, but also standardize this in the field. While not completely ignored in the literature, the understanding of multimodal communication is imperative to our understanding of the mechanisms behind linguistic, social, and cognitive development, as gesture is considered to play an integral role in the language system (e.g., Goldin-Meadow & Alibali, 2013; Vigliocco et al., 2014).

Concerning the third limitation—correlations between development and types of turn-taking cues—only a few existing studies focused on correlations between gaze, timing, and age (e.g., Augusti et al., 2010; Hilbrink et al., 2015). For instance, Augusti and colleagues (2010) showed that following a conversation is not solely linked to the interlocutors facing each other, but it also is contingent on the social norms of discourse. While Hilbrink and colleagues (2015) found that infants’ responses in terms of temporal congruency are dependent also on social development and the developmental cognitive faculties beyond turn-taking, age, or social cognitive development alone. While these are not the only two instances of these types of correlations between aspects of turn-taking, furthering our knowledge in this regard is vital to our understanding of the mechanisms behind turn-taking and social interaction. As such, as introduced in the first limitation, an open science project or consortium could foster such an endeavor. Furthermore, while there has been an extensive amount of research conducted, many claims remain unsubstantiated—such as the claim that turn-taking reaches adult-like maturity by the age of 6 years (Ervin-Tripp, 1979). To this end, extensive corpora, including multimodal and gestural interactions, and the use of corpora analysis would allow for the exploration of cue correlations and development both within and between cultures (cf. Dingemanse & Liesenfeld, 2022; Liesenfeld & Dingemanse, 2022).

The fourth limitation—cultural differences—has been investigated in a few studies (e.g., Keller et al., 2008; Lammertink et al., 2015). For instance, Keller and colleagues (2008) showed differences in the development of timing in turn-taking between two different cultures, specifically German and Cameroonian Nso. These differences indicate that the development of turn-taking, specifically in contingent timing, is dependent on the temporal structure of the cultural-specific verbal norms in dialogue. Similarly, Lammertink and colleagues (2015) revealed that English and Dutch cultures utilize different cues similarly and even, if given a forced option, prefer lexico-syntactic cues over prosodic cues similarly across the two cultures. Yet there is a lack of understanding the extent to which cultural differences affect the acquisition of turn-taking abilities in the early years of life and how these differences persist in adult conversational turn-taking. Furthermore, the diversity of cultures and languages studied is relatively small (cf. Dingemanse & Liesenfeld, 2022; Stivers et al., 2009). This aspect has been well documented by Henrich and colleagues (2010), who showed that the main body of existing research is heavily biased towards WEIRD cultures (Western, Educated, Industrialized, Rich, Democratic societies) and their languages. It is vital to address these differences as WEIRD and non-WEIRD languages may have fundamental differences, such as the differences in linguistic expression of perceptual modality within vocabularies (Majid et al., 2018) and may expand into other areas of verbal and nonverbal communication. While some research does attempt to highlight this lack of linguistic variety by including a list or map of languages and cultures included (e.g., Nguyen et al., 2022), this is often not the case. Strategies to improve cross-cultural comparisons in developmental research has been outlined and explored in detail, with specific suggestions given (Wen et al., 2025).

The fifth and final limitation—lack of universal coding scheme and cohesive study designs—has been largely addressed and outlined in papers investigating a cross-cultural stance or in the creation of new coding schemes (e.g., Gabouer & Bortfeld, 2021; Gabouer et al., 2020; Liesenfeld & Dingemanse, 2022; Wen et al., 2025). For instance, Wen and colleagues (2025) not only identified issues in cross-cultural research but also clearly stated that to improve construct validity, research must strive to improve cultural knowledge, create casual frameworks, and refine measures according to culture. Gabouer and Bortfeld (2021) have also addressed how important it is to have a universal coding scheme, which allows for expansion to other modalities. Pika and colleagues (2018) made a similar claim for turn-taking with a focus on cross-species comparisons. However, another crucial aspect to consider is the difference between laboratory experimental settings and spontaneous and natural settings. While considerations for experimental designs have long been thematized (e.g., Campbell, 1957), the question remains about how to determine which approach is more appropriate for the study. One example of this can be seen in an experiment on gaze behavior in a natural setting versus watching the same behavior in a film in a lab (Foulsham et al., 2011). While both methods produced in some regard similar results, there were differences between the two designs. This should be further considered for turn-taking in development. Currently, many suggestions aim for the maximization of the natural setting (e.g., Liesenfeld & Dingemanse, 2022), and others still suggest that an experimental design can be appropriate if construct validity has been met (Wen et al., 2025). While a deeper examination and general determination of this is beyond the scope of the current review, we would like to highlight the lack of universal coding schemes and how important study designs are to research—specifically, given the lack of cohesion between study designs. We specifically address the need for universal frameworks, such as in Wen and colleagues (2025), by presenting the framework proposed by Pika and colleagues (2018) in the section titled “Comparative Framework and the Evolutionary Trajectory,” below.

## Turn-taking comprehension

As with many aspects of language and social development (e.g., Benedict, 1979), turn-taking comprehension may precede production. Hence, we present existing studies and findings concerning turn-taking comprehension in the first 6 years of human childhood. We first begin with a brief overview of social interaction in general and then review the literature as it pertains to each fundamental concept and cue to turn-taking consecutively. Within each of the subsections, we present and summarize the findings from the literature, as well as providing critique and identifying gaps, when appropriate.

### Social interactions (in general)

Interactions between caregiver and child are important for early development on several levels, including social, emotional, and cognitive development (Cruz et al., 2020; Fogel, 1993). Beyond interactions, the coordination of interactions between those involved, in this case, infants and caregivers, are necessary by adjusting the temporal structure of a given interaction, also known as synchrony (Feldman, 2007). Furthermore, synchrony in mother–child interactions, as well as other caregiver–child interactions, has been investigated as concepts of, but not limited to, reciprocity, shared affect, attunement/mutuality, rhythmicity, harmonious interaction, and maintained engagement (Leclère et al., 2014). Synchrony in early interaction is not only a key multimodal feature of mother–infant interaction but also affects cognitive processing, linguistic features of word–object mapping and part–whole labeling, and re-pairing (i.e., the ability to persist in communication and to modify, repeat, or revise a signal when the initial communication attempt failed) interactive mismatches (Leclère et al., 2014).

Moreover, synchrony, or rather temporal coordination, is equally important for conversation and, particularly, for the timing within social events between agents. A considerable body of work on coordination dynamics provides interaction loops and defines neural cognitive mechanisms for achieving synchrony between human interactants (e.g., Oullier et al., 2008; Tognoli et al., 2020). In these models, spontaneous patterns of synchrony are formed in physical, biological, and social systems, which, in the case of synchrony and turn-taking, can be considered to be related to the principles of uni- or multimodal exchanging of information in self-organization within the coordination dynamics of the brain and behavior, shared between interactants (Oullier et al., 2008). This type of coordination of perception and action are grounded in dynamic sensorimotor loops that are shared between two or more agents (Tognoli et al., 2020). This cyclical exchange and dynamic coordination is defined in turn-taking by the oscillator model of timing (Wilson & Wilson, 2005). Neural oscillators in the activation patterns for cognitive processes of perception, motor control, memory, attention, and consciousness are used to describe a dynamic, cyclical model of synchrony in timing that is described as phase locked and also influences the listener (Wilson & Wilson, 2005). The oscillator model of turn-taking makes assumptions about the timing and readiness of the listener to respond to the speaker. The frequency of the oscillation being determined by the speaker’s syllable rate, the frequency of the oscillation determining the readiness of the listener, the listener’s cycle being counterphased to the speaker—potential to take the next turn being only at the beginning or midphase of the speaker—and in the case of a failed turn-taking sequence, the cycle only carries on for a short time before phasing out (Wilson & Wilson, 2005). This model of timing describes converging speech rates, variation of timing, phoneme identification, syllable representation, breathing rates and timing, synchronic syllable production, timing of gaps and overlaps (see “Overlaps and Gaps” section for a review), within-speaker silences, as well as simultaneous production in larger conversations (Wilson & Wilson, 2005). Taken together, synchrony in social interaction has a large role in turn-taking and can be used to model interactions by means of cyclic rhythms and coordination dynamics and have evolutionary benefits in social dynamics (Verga et al., 2023).

In infancy and childhood, social interactions (i.e., the interaction engine; Levinson, 2006, 2016), together with their synchrony (i.e., temporal coordination) are also important for the development of turn-taking (Rocissano et al., 1987). This has been shown in the studies on the development of conversation, where children by the age of 6 months are initially attracted to social components of interaction independent of auditory input (Bakker et al., 2011). The authors indicated, however, that with the increase of age, the importance of speech becomes greater when considering predictive gaze shifts in conversation. This aspect of turn-taking will be further reviewed in the “Verbal Cues” section, below.

### Timing

Previous studies on timing in turn-taking have focused mainly on production. For instance, Keller and colleagues (1999) were the first to describe 3-month-old infants considering maternal multimodal responses most likely to be contingent when produced within 1 s of the infant’s turn, be it a vocalization, gaze, or smiling, with contingent responses being produced as quickly as within 200 ms. The authors measured contingency by means of a responsiveness index and a dependency index, correcting conditional probability by means of unconditional probability, essentially baselining by frequency within a specific time window (Keller et al., 1999). Subsequently, Van Egeren and colleagues (2001) questioned this consideration of contingency by quantifying responsiveness with an improved statistical index. This was done by not only analyzing contingency across different time windows but also by analyzing two further factors: 1) signal-response combinations to assess mutual responsiveness and degrees of influence between mother and infant and between channels and 2) effects of interactional context during times of infant exploration and holding an object and during eye contact. Furthermore, the authors expanded the coding scheme to include a wider range of verbal and nonverbal actions and responses (Van Egeren et al., 2001). With this improved operationalization, 4-month-old infants judged the overall responses of the mother to be contingent when produced within 3 s of the infant’s action. However, when considering maternal vocalizations and touch in signal-response events, a latency of 2 s best defined contingency, while nonverbal responses such as smiling were considered to be contingent for up to 3 s (Van Egeren et al., 2001).

Another window into timing in turn-taking and the evaluation of contingent responses is to measure brain responses to contingent and noncontingent mother–infant interactions. For instance, Lam-Cassettari and colleagues (2021) investigated infants’ (6- and 9-month-olds) brain responses of contingent (< 1.5 s) and noncontingent (> 2 s) caregiver responses to infant utterances. The event-related potential (ERP) analysis of the 6-month-olds yielded an increased positive response (P3), from 100 to 200 ms after the onset of the stimuli, for the contingent over the noncontingent responses. These results indicated that the 6-month-old infants showed increased attention and arousal for the contingent condition. In contrast, the ERP analysis of the 9-month-olds yielded a negative response (N400) to contingent caregiver stimuli, indicating the processing of conceptually meaningful stimuli (Lam-Cassettari et al., 2021). Importantly, the authors interpreted these results as the 6-month-olds being sensitive to contingent responses of both infants and caregivers, while 9-month-olds were more sensitive to contingent caregiver responses. Only the negative responses of the 9-month-old infants were interpreted as the elicitation of lexico-semantic access (Lam-Cassettari et al., 2021).

While these three studies have provided age-related developments on the comprehension of timing in turn-taking sequences, it is important not to lose sight of the larger picture. Due to the differences in methodology, there is considerable uncertainty concerning the comprehension of contingent versus noncontingent utterances. Keller and colleagues (1999) did consider times beyond 1 s, but described contingent as within 1 s. However, there were also reactions that occurred at later time points. Van Egeren and colleagues (2001), on the other hand, were much systematic in their testing of what is considered “contingent,” reliant on the type of action taken by the infant and the specific response type of the mother. While reaction to contingent responses in general are described as occurring as early as 3 months of age, it is important to look into the methodology behind the findings. Furthermore, the labeling of contingent and noncontingent vocalizations by Lam-Cassettari and colleagues (2021) is based on the findings of Van Egeren and colleagues (2001). Despite this, the methodological goal behind the ERP analysis went beyond the specific timing of contingency, focusing on the neurocognitive mechanisms behind the comprehension of timing. Although the reviewed studies provide insight into the processing and comprehension of timing during turn-taking, how infants comprehend variations of timing between turns has not been systematically assessed across development. Consequently, a detailed understanding of the comprehension of standard timings during development is lacking. Furthermore, it is still difficult to pinpoint a clear definition of contingent and noncontingent responses, as absolute upper boundaries have not been defined. These definitions are modality-specific (Keller et al., 1999; Van Egeren et al., 2001) and may depend on other factors beyond developmental age and turn-taking, such as social cognition and language development. Furthermore, contingency may also be affected by differences in speech type, such as infant-directed versus adult-directed speech, which has not been directly tested.

### Gaze

Eye gaze is a crucial factor in development because it is vital for sophisticated social cognitive skills such as joint attention, the orientation of attention, and facial recognition (Frischen et al., 2007). In addition to social cognition, eye gaze is important for the development of language in domains such as vocabulary, word–object mappings, object processing, and speech processing (Çetinçelik et al., 2021). Different methodologies have been used to measure gaze, including head-turn paradigms, preferential looking (including both familiarity and novelty effects), and eye-tracking, which includes gaze-tracking, pupillometry, and blink rate (Eckstein et al., 2017).

For the comprehension of turn-taking, this section focuses on gaze direction of the infant as well as the two interlocutors that the infant watches in third-person observational studies. Guellai and Streri (2011) were able to show that newborn infants were able to recognize individuals who have previously spoken to them only in situations where the individuals directly looked at the infant, but not in situations where the individuals looked away. Hence, the results suggest that facial recognition in early infancy relies on gaze and plays a crucial role for social interaction. This can further be seen when considering the ability of infants to extract temporal information from a social interaction in one modality and, in turn, transfer this information to a different modality (Crown et al., 2002). Crown and colleagues (2002) provided evidence that 6-week-old infants were able to coordinate their gaze to both maternal and stranger vocalizations. Again, possible differences between infant-directed speech and with adult-directed speech were not investigated. While adult gaze provides information to neonates that they are involved in social interaction, infant gaze is not only coordinated to adult vocalizations but also provides information to adults, which can be used to regulate pauses in conversation (Crown et al., 2002). However, when processing conversations as third-party observers, infants also utilized gaze as a cue to turn-taking. In 6-month-olds, gaze to the second speaker in a turn-taking sequence in the case of overlaps (see “Overlaps and Gaps” section for more information) was influenced by the socioeconomic status (SES) of the infants’ families (Meunier et al., 2024). While low and middle-low SES infants’ gaze patterns did not differ between turn-taking, giving the turn, and overlap sequences, the gaze patters of middle-high and high SES infants showed higher rates of looking to the second speaker in overlap situations (Meunier et al., 2024). As such, the development of gaze in the comprehension of turn-taking is influenced by SES.

When processing events, the existing studies showed that 1-year-olds prefer to look at the speaker during videos of a conversation rather than the nonspeaker; however, the 3-year-olds were able to predict turn-taking by means of anticipatory gaze shift before the interlocutor began speaking (von Hofsten et al., 2009). Anticipatory gaze shifts are indicated when the participant shifts their gaze from the speaker to the interaction partner before turn-taking has occurred; in contrast, reactive gaze shifts are when participants first shift their gaze to the new speaker after turn-taking has occurred. In line with anticipatory gaze shifts, Augusti and colleagues (2010) were able so show that already at 6 months of age, infants were statistically more likely to reactively shift their gaze between interlocutors when the conversational partners were facing one another rather than facing opposite directions. However, when both conversational partners were facing in the same direction (either both facing to the left or to the right), 6-month-olds were still able to shift between the interlocutors (Augusti et al., 2010). Taken together, these findings provide evidence that infants’ comprehension of social interaction is not being primarily guided by interaction partners directly facing one another, but rather that infants’ processing of adult gaze in a social interaction is contingent on at least one interlocutor facing the other. These important findings, however, should also be taken with a grain of salt. The properties of speech were not additionally manipulated in this study, leaving an open question as to the extent of intonation, word choice, syntactic structure, vocal quality have on gaze shifts (e.g., infant-directed speech and adult-directed speech) and SES affect the development of gaze as a measure of turn-taking comprehension during childhood. In addition to being measured as the sole indicator of comprehension, gaze can also be measured in the manipulation of other cues of comprehensive turn-taking, which can be more clearly seen in the prosodic, verbal, and gestural cues to turn-taking and will be discussed in turn below.

### Prosodic cues

Already at birth, neonates are sensitive to the rhythmic pattern and prosodic structure of their native language (Demany, 1982; Demany et al., 1977). Furthermore, neonates are able to distinguish languages of different rhythmic classes (Nazzi et al., 1998), and by the age of 5 months they are able to distinguish between languages of the same rhythmic class and even between native and nonnative variants of their native language (Jusczyk, 2002; Nazzi et al., 2000). The role of prosody in turn-taking encompasses nonverbal aspects of speech, such as intonation, speech volume, speaking rate, and timber (Skantze, 2021), and has been identified as not only being important for turn-taking, but for language development in general (Gerken, 1996). Moreover, it is known that prosody marks a turn end in natural conversation by boundary cues (Gerken & McGregor, 1998).

In a study with 6-, 12-, 24-, and 36-month-olds and an adult control group, anticipatory gaze shifts in turn-taking was analyzed by presenting normal and flattened intonation in adult-directed speech (ADS), accompanied with a video of the conversation (Keitel et al., 2013). Keitel and colleagues (2013) determined that 3-year-olds were first able to reliably shift their gaze in anticipation of turn-taking, but that the younger age groups’ perception of the conversation were not influenced by the manipulation of prosody. These findings were replicated by Keitel and Daum (2015) using the same procedure with puppets, where again 3-year-olds, but not 1-year-olds, were able to reliably shift their gaze before the start of the next turn. However, this changes when children are presented with infant-directed speech (IDS). In a turn-taking study with puppets, an experiment was able to show that 1-year-olds were able to reliably predict turns when the prosodic information of the IDS conversation were complete and not ending a vocalization with unnatural intonation (incomplete intonation); yet 3-year-olds were able to reliably predict turns in both IDS and ADS (Kalashnikova & Kember, 2020). Thus, children are sensitive to the register of speech and this influences their ability to reliably predict turns in conversation. Prosody, however, is only one acoustic cue to turn-taking. Verbal cues also provide information and can influence turn-taking.

### Verbal cues

In addition to the acoustic information provided by prosody, the syntactic, lexico-semantic, and pragmatic information provided by language in conversation is a vital cue to turn-taking (Skantze, 2021). Studies looking into the comprehension of verbal cues of turn-taking in development tend to use eye-tracking methods to measure the number of gaze shifts, particularly anticipatory gaze shifts, as with the prosodic cues. Thorgrimsson and colleagues (2015) showed that both 1- and 2-year-olds shifted their gaze quicker when video conversations consisted of speech sounds than nonspeech human auditory sounds (i.e., repeated syllables, throat clearing, and vocal sighs). Furthermore, although 2-year-olds were able to produce more reliable anticipatory gaze shifts in the face-to-face condition with speech vs non-speech, 1-year-olds were able to reliably predict turn-taking when the conversational initiator more saliently marked their turns by a visual and audible inhale 2 s before their utterance (Thorgrimsson et al., 2015). Similarly, Lammertink and colleagues (2015) were able to show that both Dutch and English 2.5-year-olds made the most anticipatory gaze shifts in conversational videos with puppets when both prosodic and syntactic cues were presented completely. Participants also successfully and reliably predicted turns when syntactic information was complete but prosodic information was not complete; however, with complete prosody and incomplete syntax, participants did not perform above chance (Lammertink et al., 2015). It is important to note that prosodic cues and verbal cues are greatly intertwined, and an early advantage for one cue over the other is not found (Casillas & Frank, 2017).

In addition to complete versus incomplete syntax, other verbal cues also influence the comprehension of turn-taking in infancy. When considering linguistic cues as a whole, the type of utterance, or rather, the type of conversation, can also lead to processing differences in development. Casillas and Frank (2017) showed that there is a *question effect* during the first years of life. The question effect describes the finding that both children (ages 3–6 years) and adults made more anticipatory gaze shifts when questions were asked than when non-question conversations were presented (Casillas & Frank, 2017). This supports the notion that spontaneous turn-taking predictions may not simply be the prediction of the ending of a turn, but rather what occurs thereinafter (Bögels & Torreira, 2015; Casillas & Frank, 2017; de Ruiter et al., 2006; Keitel et al., 2013; Magyari & de Ruiter, 2012). However, interactions between the question effect and IDS in the case of turn-taking have not yet been explored. Although prosodic and verbal cues seem to be largely intertwined in development, both are important cues to turn-taking and develop independently from one another.

### Gestures

Nonverbal communication, in the form of gestures, is also important for social interaction, language learning, and language use (Goldin-Meadow & Alibali, 2013; Vigliocco et al., 2014). In adult turn-taking, response times became quicker when gestures are used while asking questions (Holler et al., 2018). Developmental studies also showed that mothers react to infant vocalizations with gestures and movement (Lüke et al., 2017; Reddy et al., 2013). As early as 2 months of age, infants anticipate physical interaction while mothers reach for them to pick them up, and this coordination becomes smoother over time (Reddy et al., 2013). Furthermore, infants are sensitive to delays in being lifted up after mothers have already touched the infant (Fantasia et al., 2016). These anticipations show that infants comprehend and react to nonverbal cues to turn-taking in a social interaction. During third-party observation, 14-month-olds were found to anticipate specific reactions to a conversational partner’s gestures (Thorgrimsson et al., 2014). Taken together, infants both react to nonverbal turns and expect others to react to gestures in third-party observations.

## Turn-taking production

This section addresses turn-taking production with a special focus on timing, overlaps and gaps, repairs, gaze, prosodic cues, verbal cues, and gestures. Overlaps and gaps are considered together based on how the two functional concepts are considered and studied in the developmental literature.

### Social interactions (in general)

Infants participate in social interaction involving turn-taking even as neonates (Nagy, 2006; Nagy & Molnar, 2004). This productive form of interaction, as described in the “Social Interactions (In General)” section, is also influenced by synchrony in social dynamics, coordination dynamics, and the oscillator model of turn-taking (Leclère et al., 2014; Oullier et al., 2008; Tognoli et al., 2020; Wilson & Wilson, 2005). Although infants participate in turn-taking shortly after birth, turn-taking continues to develop over the next 6 years of life (Ervin-Tripp, 1979), despite the lack of further evidence to when turn-taking skills have been completely acquired or are fully developed. In these first interactions, it is also seen that infants modify their movements to external rhythmical stimulation (Provasi et al., 2014).

Even later in childhood, mother–infant conversations are largely managed by the mother, providing evidence that even in the third year of life, children assume a reciprocal role rather than an imitative role (Kaye & Charney, 1981). However, when 3-year-olds talk to each other, children are capable of having a conversation by repeating and modifying the utterances of their interlocutor, also referred to as sound play, and a conversational structure can be abstracted from these interactions (Keenan, 1974). There is also variability in how mother–infant communication presents itself between cultures. German mothers seem to produce more temporally congruent responses to infant vocalizations more than mothers in a small village in Cameroon (Keller et al., 2008), although the validity of what is considered to be contingent is somewhat uncertain. It should also be noted that much of mother–infant interactions are largely investigated by looking at a targeted mode of communication instead of a holistic, multimodal approach (Levinson, 2024; Rohlfing et al., 2020). This limits our knowledge of the development of turn-taking and social interactions, as past experiments and observations may have not included the use of gesture, verbal, IDS and ADS, or even the combination thereof in the development of turn-taking production.

### Timing

The existing studies on the development of timing during turn-taking production have focused on vocalizations, interaction, and their temporal contingency. For instance, Jasnow and Feldstein (1986) showed that 8.5-month-olds not only respond in a similar temporal latency to mothers’ responses, but that the infants are influenced by the current temporal latency behavior of the mothers. Similarly, Dominguez and colleagues (2016) demonstrated that responses of neonates (2–4 days old) to maternal vocalizations occurred within 1 s 68.9% of the time and were latched (produced within 50 ms of maternal vocalizations) 26.9% of the time. In a more detailed study by Hilbrink and colleagues (2015), a shift in infant response latency was discovered. The researchers found that around 9 months of age, infant responses became more delayed than in previous months but stabilized and returned to faster responses around 18 months of age (Hilbrink et al., 2015). Not only was this increase in response latency at 9-months confirmed by Smith and McMurray (2018) but the authors also found that overall infant response latencies decreased between four and 60 months of age. These increases in latency at 9 months are indicative of the integration of these linguistic skills into the interaction engine (Hilbrink et al., 2015; Levinson, 2006, 2016; Smith & McMurray, 2018). However, these findings just contribute two examples of a much greater body of research. Nguyen and colleagues (2022) conducted a meta-analysis on the development of timing in turn-taking between adults and children in vocal interactions. The authors found weak evidence that responses first begin to slow down around 20 months, not 9 months. However, this claim is difficult to support due to methodological differences between many of the included studies. A recent study has found that between 24 and 60 months, children increase the speed of their responses between 28 and 46 ms every 4 months (Fusaroli et al., 2024).

Children from 1 year of age are quickest to answer simple yes/no questions, but response latency for more complex questions increases (Casillas et al., 2016). Furthermore, the authors were able to show that response latencies of more complex questions shortened overtime to the age of 6 years (Casillas et al., 2016). Lindsay and colleagues (2019) found that preschoolers (3- to 5-year-olds) were able to predict the end of a question and were able to optimize their timings, producing rapid responses while still learning complex language. Both of these findings fall in line with the question effect (see “Verbal Cues” section, above; Casillas & Frank, 2017), indicating that there temporal advantages in both comprehension and production when children are addressed with a question. Although the reported timings of each of the studies may vary, it seems that children are able to produce contingent responses to maternal vocalizations from birth and become faster in their responses over the first 6 years of life (for a more detailed review on the timing of infant vocalizations through development including atypical populations, see Nguyen et al., 2022). Despite these findings, open questions again include the effect that IDS has on the timing of production for children, while it is also unknown to what extent modality also would play a role in timing of children’s responses (for similar arguments on the effects of modality on language, see, e.g., Bechtold et al., 2023; Cosper et al., 2020, 2022).

### Overlaps and gaps

In this section, three aspects of turn-taking are considered: contingent responses (gaps longer than 50 ms but below the noncontingent threshold; see the “Timing” section for a more detailed criticism of contingency), latched responses (produced between 0 and 50 ms of maternal vocalization, but not temporally overlapping), and overlaps (where both mother and infant produce vocalizations at the same time, also known as co-action) without focusing on the response latency in detail. While Sacks and colleagues (1974) describe one of the facts of turn-taking being to minimize overlaps, it is important to note that overlaps contribute to fluent conversations and provide important functions (Coates, 1994; Heldner & Edlund, 2010; Skantze, 2021). Ginsburg and Kilbourne (1988) provided first evidence that infant turn-taking first shifts from a co-action (overlapping) vocalization pattern to a proto-conversation in the second year of life. However, Stern and colleagues (1975) described the vocalization pattern of infants to work in both in co-action (overlapping) as well as alternating (gap); although the authors do agree that only in the second year of life would infant vocalization patterns predominantly shift to an alternating pattern.

More recently, Gratier and colleagues (2015) assessed gaps and overlaps in mother–infant interactions. The authors found that even at 2–3 months of age, infant vocalizations were preceded by maternal vocalization (either with a gap or latched vocalizations) at a higher rate than overlapped. This pattern is also still present in many interactions involving 4-to-5-month-olds (Gratier et al., 2015). A longitudinal study, however, provided evidence that overlaps decrease between 5 and 18 months of age to only occurring in a fifth of turns (Hilbrink et al., 2015). Although, a more recent longitudinal experiment indicates there is little change in the frequency of overlaps between 20 and 40 months, but the duration of the overlaps does decrease (Fusaroli et al., 2024).

In sum, these studies suggest that a shift from a more dual vocalization pattern of infant turn-taking to a more alternating pattern already takes place in the first year of life but become more stable in the second year of life. This can furthermore be explained by the importance of synchrony but also by the models of coordination dynamics and the oscillator model of turn-taking. Over the course of the first year of life, infants become more active in social interactions. This could be considered to be the influence of synchrony in social coordination dynamics, where the infant and the mother affect one another and begin to link on a dynamic loop (Oullier et al., 2008; Tognoli et al., 2020). Furthermore, the oscillator model of turn-taking can also be used to describe the decrease in the duration of overlaps in productive turn-taking over the course of the second and third years of life (Fusaroli et al., 2024; Wilson & Wilson, 2005). Although the frequency of overlaps did not decrease, the reduction in overlap time may be an indication that children are beginning to develop shared cycles of timing and are influenced by them in turn-taking exchanges. How and when these processes develop and are fully developed is currently unknown. Despite this, the influence of these systems on the development of turn-taking can be assumed and may provide crucial research avenues for future research.

### Repairs

In language production, it is possible for mistakes to occur (cf. Sacks et al., 1974). Clark (2020) provided a review on the development of prompted (other-initiated) and unprompted (self-initiated) repairs from children as well as restricted offers (corrections from adults in adult–child interactions). While linguistic repair is important to communication and language development, repairs in turn-taking refer mainly to the alternating structure of the conversation (cf. Sacks et al., 1974). Although repairing turn-taking errors and violations to reinstate the cooperative interaction and the communicative flow is an important functional concept of turn-taking, we know relatively little about the development of communicative repair within productive turn-taking in childhood. Garvey and Berninger (1981) noted that children from the age of 2;10 to 3;3 years when conversing with peers already notice when their interlocutor missed a turn and accordingly attempted to repair the error. However, the authors did not investigate multimodal cues to this as the coders were intentionally not trained to identify specific movements (Garvey & Berninger, 1981). Gallagher and Craig (1982) investigated triadic conversations between 4-year-old girls and found that the three conversational partners were able to manage their conversation and prematurely ended turns when sentence-initial overlaps occurred. In addition, a study on mother–child interactions showed that infants ages 34 to 75 months of age ended overlaps at a higher rate (51.2%) than the mothers (22%). Furthermore, in mother-initiated overlaps, children also discontinued their turns proportionately more often (66.7%) than did the mothers (16.7%; Bedrosian et al., 1988). Taken together, these findings show that children are able to recognize violations to turn-taking and actively implement repair mechanisms to attempt to correct them already at the end of the second year of life. Sacks and colleagues’ (1974) statement still holds true today, at least for the development of conversational turn-taking; few studies focus specifically on the functional concept of repair in turn-taking violations during development.

### Gaze

Infants gaze at their mothers already at the age of 1 month, and gaze rates increase during mother vocalizations at the age of 3 months (Henning et al., 2005). These findings emphasize how infants become more active in social interactions and gaze develops already between 1 and 3 months. Rochat and colleagues (1999), however, used an organized versus disorganized peek-a-boo experiment, showed that while 2-month-olds maintained gaze with a stranger during an organized social interaction for longer periods of time, 4-month-olds looked at the social partner significantly less often. The authors attribute this shift to an emerging sensitivity to the timing and structure of the social interaction and less on the interaction partner (Rochat et al., 1999). Furthermore, Rutter and Durkin (1987) provided evidence that even though child gaze to mothers most frequently occurs when mothers are vocalizing, children from the age of 18 months begin to reliably use the *terminal look* at the end of a mother’s turn, checking for signals marking turn-taking and the child being able to take the floor. In three-party interactions between 4-year-olds, while child gaze is typically focused on self or a single other child, the speaker used gaze before the end of their turn to look at another child, which was most frequently the next speaker (Craig & Gallagher, 1982). As such, young infants use gaze to indicate social engagement, while older children gaze less often in order to focus on other attributes of the social interaction. Moreover, in the second year of life, infants use terminal look to check for turn-taking signals, and older children (4-year-olds) are able to use gaze to indicate which interaction partner should take the next turn in a multiparty interaction.

### Prosodic cues

Although prosodic cues are important in infant turn-taking comprehension, such as voice quality, IDS versus ADS, quicker responses to questions, and more, relatively little research has been conducted on how the development of infants’ prosodic cues affect productive turn-taking and when and how infants implement prosodic cues in turn-taking sequences. A single study showed that infants were able to imitate the prosodic contour of mother vocalizations already at the age of 3 months (Gratier & Devouche, 2011). Thus, there is much yet to be explored in infants’ use of prosodic cues as they pertain to turn-taking and how they develop.

### Verbal cues

In this section, infant vocalizations in terms of syntactic, lexico-semantic, and pragmatic cues are reviewed. Already as neonates, 3-day-old infants were more likely to produce vocalizations in the presence of maternal vocalizations than without them (Rosenthal, 1982). This result is in line with the findings presented in the section above on “Gaps and Overlaps.” Concerning the quality of vocalizations, in the third month of life, infants produced more speech-like sounds than non-speech-like sounds when the adult conversation partner maintained a give-and-take vocal interactional pattern (Bloom et al., 1987). Moreover, the rate of infant speech-like vocalizations was only higher when adult vocalizations were speech sounds than when they were nonspeech sounds (Bloom, 1988). This finding does not provide evidence that adult turn-taking behavior facilitates verbal development, but rather infants preferred to react to and respond to verbal rather than nonverbal vocalizations. This may suggest that turn-taking sequences draw more attention of children, eliciting more speech-like sound responses (Bloom, 1988; Bloom et al., 1987). Similarly, in a still-face paradigm, it was shown that 5-month-old infants were aware that their vocalizations in the form of cries had an effect on their social interaction partners, when the partner was a stranger, while infant smiles did not show a similar excitement burst from a stranger (Goldstein et al., 2009).

In the beginning of the second year of life, children also produced more vocalizations in a mother–child game of peek-a-boo after the object/subject reappeared rather than before; however, the absence of a vocalization was still more common than the presence of vocalizations (Ratner & Bruner, 1978). In a longitudinal study, Rome-Flanders and Cronk (1995) identified that infants produced several types of vocalizations in both a ball game and peek-a-boo with their mothers. However, one type of vocalization, a single vowel or sequence of vowel sounds (referred to by the authors as primitive vocalizations), was more frequently produced in the ball game than in peek-a-boo and only slightly changed in absolute frequency over time while its relative proportion declined (Rome-Flanders & Cronk, 1995). The authors argued that children between the ages of 0;6 to 2;0 years use this type of vocalization in the ball game, although other linguistic structures were available to them; the authors attributed this to the vowel sounds being used as a pragmatic indication of willingness to participate, while in peek-a-boo the children relied on linguistic expressions (Rome-Flanders & Cronk, 1995).

Donnelly and Kidd (2021) found in a longitudinal study that there was a bidirectional relationship between vocabulary growth and conversational turns between the 9th and 24th month of life. Furthermore, the authors suggested that infants were able to jointly control the interaction engine, indicated by an active involvement in the acquisition process—increases in linguistic development allow for children to facilitate their own turn-taking development (Donnelly & Kidd, 2021). Overall, these findings suggest that with age, infants are able to utilize their vocalizations in a more targeted manner and the relationship between turn-taking development and verbal development appears to be bidirectional.

### Gestures

Gesture production, as a cue to turn-taking, has not been considerably studied. However, insights from this communicative modality can help us further understand how turn-taking develops in infancy and childhood, and better understand the development of social conversation (Rohlfing et al., 2020), as gesture and multimodal communication are inherently linked to language and therefore turn-taking (Goldin-Meadow & Alibali, 2013; Vigliocco et al., 2014). Even shortly after birth, infants were able to imitate specific facial expressions and also initiate the alternating social interaction with accelerated heart rates for initiation and decelerated heart rates for imitation (Nagy, 2006; Nagy & Molnar, 2004). In a study by Nomikou and colleagues (2017), infants at the age of 4 months actively participated in peek-a-boo games with their mothers, and already attempted to uncover themselves or their mother, assumingly to actively participate in a motoric fashion. Although the infants were not always successful, the action can be considered to be a multimodal form of participation in the turn-taking sequence of peek-a-boo. Furthermore, the infants reacted with smiles and movements after the uncovering phase of the peek-a-boo game (Nomikou et al., 2017). At 6 months, infants’ attempts to uncover, and their smiles as a result of the uncovering, increased significantly, indicating that active participation in the coordinated interaction increased with age (Nomikou et al., 2017).

Although the studies reviewed above show active participation in social routines by the use of gestures and multimodal interaction, it is important to understand how productive gestures and language are intertwined in development (Goldin-Meadow & Alibali, 2013). For instance, the development of and the rate of using the pointing gesture in the second year of life can be an indication of language development (Lüke et al., 2017). Lüke and colleagues (2017) provided evidence that at 12 and 14 months of age, children with a language delay produced fewer index-finger pointing gestures (as opposed to using hand pointing gestures) than typically developing children at the same age. Furthermore, children with a language delay reached the milestone of index-finger pointing around 2 months later than typically developing children (Lüke et al., 2017). In turn-taking, the same pattern can be seen in a case study about answering *Where* and *Which* questions with a gesture and/or a vocalization (Clark & Lindsey, 2015). The authors reported that across monthly video data from the age of 1;5 to 3;5, the subject in question overall answered *Wh-* questions significantly quicker with a gesture than with a babble, repeated word, or a new word. Furthermore, in combined answers, the analysis showed that gestures were still produced much quicker than the verbal response (Clark & Lindsey, 2015). However, response times increased with the complexity of the question and also varied across response types (Clark & Lindsey, 2015). This is in line with the development of verbal responses to questions as it pertains to the complexity of the question (Casillas et al., 2016) and the question effect (Casillas & Frank, 2017).

## Milestones of turn-taking in development

Currently, the developmental trajectory of turn-taking and the acquisition of turn-taking functional concepts and cues is not clearly understood, particularly also concerning a multimodal perspective (Levinson, 2024). Hence, the second aim of this multifaceted review is to identify possible milestones in the development of human turn-taking, based on the literature review presented in the “Turn-taking Comprehension” and “Turn-Taking Production” sections. In order for a milestone to be determined, we only focused on the first evidence of the comprehension or production of a particular aspect of turn-taking. As such, many of these proposed milestones have not been validated or corroborated by other studies, unless otherwise indicated by multiple sources in the text and tables. These milestones should be understood as tentative only and entirely based on the current literature. However, we hope to instigate more interest into this field of research with future studies testing, verifying, or adjusting these milestones.

It is important to additionally note the uncertainty of how methodological choices and statistical decisions have affected the suggested milestones. We have taken the authors at their word within the context of their given statistical analyses. This, however, can affect the interpretation of the results the subsequent identification as a possible milestone or even the exclusion of potentially valid milestones. Possible factors that could influence the identification of milestones include differences in methodologies, statistical analyses, assumptions, sample sizes, cultures, and study designs (i.e., cross-sectional or longitudinal designs). Methodology can influence milestones due to differences in operationalizations. As such, when discussing reactive versus anticipatory gaze shifts, the method in which saccades are measured can be crucial—accuracy between eye-tracking studies and video-recordings could possibly differ, leading to the misidentification of a milestone. Beyond operationalization, the base assumptions within a study can further influence the identification of developmental milestones. Considering the debate on contingency in in the “Timing” section, assumptions based on verbal and nonverbal expressions and statistical measures lead to varied results (cf. Keller et al., 1999; Van Egeren et al., 2001). Furthermore, the statistical analysis could also influence the precise identification of a milestone—how differences between conditions was measured and which additional factors (be it fixed factors as in a mixed-effects linear model, covariates in other measures, or the use of Bayesian statistics) may have influenced statistical thresholds across studies. Another important factor is the sample itself. One influential factor is the sample size, which can influence the ability to identify large, robust effects (Brysbaert, 2019). Culture is another sample-based factor that can influence the identification of milestones, including lack of cultural knowledge, lack of causal frameworks, and culturally inappropriate tools and measures (Wen et al., 2025).

Finally, study design may also affect the identification of developmental milestones. While cross-sectional study designs can show differences between selected age groups, these age groups are largely limited and may not cover all ages relevant for a milestone to be identified. Furthermore, a cross-sectional study cannot provide information on individual development as with a longitudinal design. Longitudinal studies also take more time and resources, thus limiting our current understanding based on the available data from the current literature.

We caution the readers and strongly suggest longitudinal studies and meta-analyses for the conformation of any and all proposed and the identification of milestones not proposed. While a quantitate synthesis of the current literature or a meta-analysis could yield a more fine-grained overview of the literature, it was beyond the scope of the current review to conduct such an analysis. As our identification of milestones is thereby limited, we strongly urge this to be done in the future. In the subsequent paragraphs, we therefore provide a first preliminary set of milestones for turn-taking comprehension and production linked to developmental age.

**Fig. 1:**
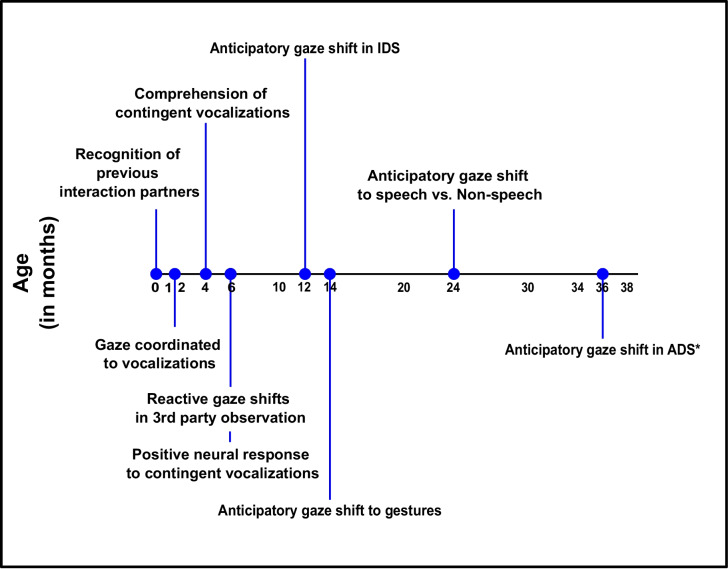
This figure provides a timeline, in months, and specifies the milestones of comprehensive turn-taking. Validation of a milestone by means of corroboration in the literature is marked by a (*)

Concerning the development of comprehensive turn-taking, infants begin as neonates with the ability to gaze at and recognize individuals who have spoken to them before and looked at them (Guellai & Streri, 2011). At 1.5 months, infants were able to coordinate gaze to both maternal vocalizations as well as to vocalizations of strangers (Crown et al., 2002). At 4 months of age, infants considered maternal verbal responses to be contingent within 2 s and nonverbal responses to be contingent within 3 s (Van Egeren et al., 2001). At the age of 6 months, infants have two milestones. First, 6-month-olds were able to reliably produce reactive gaze shifts between two interlocutors in a conversation when the two conversational partners are facing each other than when facing opposite directions (Augusti et al., 2010). Second, 6-month-olds showed a positive brain response to contingent versus noncontingent responses between 100 and 200 ms after the onset of the vocalization; furthermore, at 9 months of age, infants had a negative response around 350 ms after the onset of a vocalization, indicating a sensitivity to the lexical-semantic information of the utterance, whereas 6-month-olds were sensitive to the timing of the response (Lam-Cassettari et al., 2021). At 12 months, infants were able to reliably predict turn-taking by means of anticipatory gaze shifts, when conversation was presented in IDS (Kalashnikova & Kember, 2020). Similarly, children at 14 months of age were able to produce anticipatory gaze shifts to gestures in third-party observation (Thorgrimsson et al., 2014). At the end of the second year of life, 24-month-olds were more able to reliably produce anticipatory gaze shifts to speech than nonspeech when conversation was presented face to face (Thorgrimsson et al., 2015). Finally, at the end of the third year of life, 36-month-olds were able to anticipate turn-taking in ADS (Kalashnikova & Kember, 2020; Keitel et al., 2013). All information is presented in table form (see Table 3) and graphically (see Fig. 1). We hope that the presented information can inform future cross-sectional and longitudinal studies to verify, extend, or challenge these milestones and expand them to individual development. Furthermore, a secondary future goal may be to validate, challenge, and expand these milestones and others across cultures, beyond WEIRD societies, by means of corpora analysis and meta-analyses.

**Table 3:** Chronological development of skills and aspects related to comprehensive turn-taking

Author/Year	Age	Milestone
Guellai and Streri (2011)	Neonate (50.5 h)	Recognition of previous interaction partners (via gaze)
Crown et al. (2002)	1.5 months	Gaze coordinated to vocalizations, both familiar adults and strangers
Van Egeren et al. (2001)	4 months	Comprehension of contingent vocalizations (within 2 s)
Augusti et al. (2010)	6 months	Reactive gaze shifts to turn-taking patters during 3rd-party observation
Lam-Cassettari et al. (2021)	6 months	Positive neural response to contingent vocalizations vs. noncontingent (100–200 ms)
Kalashnikova and Kember (2020)	12 months	Anticipatory gaze shift to turn-taking patterns in 3rd-party observation when speech is in IDS
Thorgrimsson et al. (2014)	14 months	Anticipatory gaze shift to turn-taking patterns in 3rd-party observation to gestures
Thorgrimsson et al. (2015)	24 months	More reliable anticipatory gaze shift to turn-taking patters in 3rd-party observation to speech than to nonspeech
Kalashnikova and Kember (2020) Keitel et al. (2013)	36 months	Anticipatory gaze shift to turn-taking patterns in 3rd-party observation when speech is in ADS

**Fig. 2:**
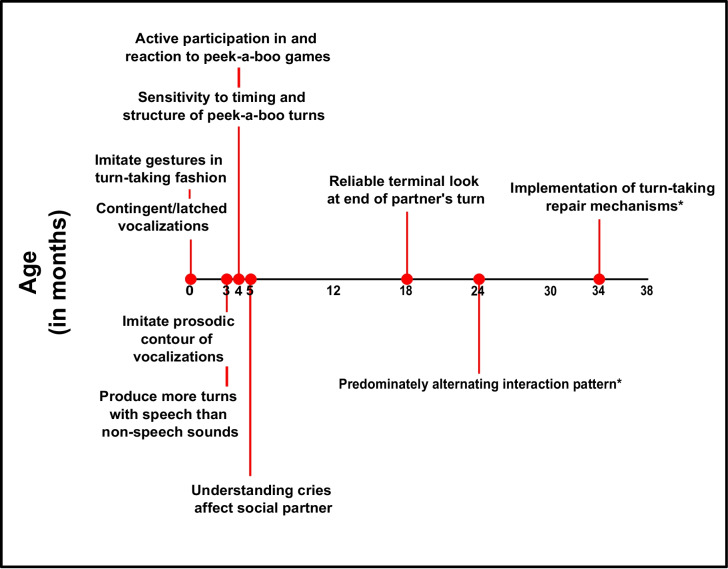
This figure provides a timeline, in months, and specifies the milestones of productive turn-taking. Validation of a milestone by means of corroboration in the literature is marked by a (*)

Concerning the development of productive turn-taking, many aspects of turn-taking are already present in neonates. First, in the first month of life, infants’ responses to maternal vocalizations were mostly produced within the first second (both contingently and latched; Dominguez et al., 2016). Secondly, neonates were able to imitate gestural turn-taking (sticking out their tongues) and initiate alternating social interaction (Nagy, 2006; Nagy & Molnar, 2004). Three-month-olds were able to imitate the prosodic contour of maternal vocalizations in alternation (Gratier & Devouche, 2017). Furthermore, 3-month-olds produced more speech-like than non-speech-like sounds in an alternating pattern maintained by adults, and these speech-like sounds were produced only at a higher rate when adults also produced speech-like sounds (Bloom, 1988; Bloom et al., 1987). In peek-a-boo games, 4-month-olds indicated sensitivity to the timing and structure in organized versus disorganized games than to gaze of the interaction partner (Rochat et al., 1999). Furthermore, 4-month-olds also actively participated in peek-a-boo games by attempting to uncover themselves and smiling as a reaction to the reveal phase of the interaction (Nomikou et al., 2017). At 5 months of age, infants showed an understanding that their cries affect their social partner in interaction, but the same level of excitement is not reached through smiling (Goldstein et al., 2009). Furthermore, 18-month-olds reliably produced a terminal look to their interaction partner towards the end of a vocalization to search for turn-yielding cues (Rutter & Durkin, 1987). Research indicated that 24-month-olds shift from a dual co-action and alternating interaction pattern to a predominately alternating pattern (Ginsburg & Kilbourne, 1988; Stern et al., 1975). Finally, at 34 months of age, first indication of the implementation of turn-taking repair mechanisms have been identified in both mother–child dyadic and peer–triadic interactions (Bedrosian et al., 1988; Garvey & Berninger, 1981). All information for the milestones of productive turn-taking is presented in table form (see Table 4) and graphically (see Fig. 2). As with the milestones presented for the comprehensive turn-taking, these developmental milestones have been identified in order to be verified, challenged, and/or expanded in future investigations. We hope that this overview may guide cross-sectional and longitudinal analyses as well as corpora analyses aimed at cross-cultural similarities and differences.

**Table 4:** Chronological development of skills and aspects related to productive turn-taking

Author/Year	Age	Milestone
Dominguez et al. (2016)	Neonate (2–4 days)	Infant responses to maternal vocalizations are contingent/latched (produced within 1 s)
Nagy (2006) Nagy and Molnar (2004)	Neonate (3–54 h)	Imitation of gestural turn-taking and alternating social interaction
Gratier and Devouche (2017)	3 months	Imitation of prosodic contour of maternal vocalizations
Bloom (1988) Bloom et al. (1987)	3 months	Infants produce more turns with speech-like than nonspeech sounds, when adult participants do the same
Rochat et al. (1999)	4 months	Sensitivity to timing of peek-a-boo turns and prefer organized game structures to disorganized
Nomikou et al. (2017)	4 months	Infants actively attempt participation in peek-a-boo games and react at appropriate phases
Goldstein et al. (2009)	5 months	Understanding that cries affect social partner and smiling does not reach the same level of excitement
Rutter and Durkin (1987)	18 months	Reliable terminal look at end of social partner’s turn to assess for additional cues
Ginsburg and Kilbourne (1988) Stern et al. (1975)	24 months	Switch from dual coaction interaction pattern to a predominately alternating pattern
Bedrosian et al. (1988) Garvey and Berninger (1981)	34 months	Implementation of turn-taking repair mechanisms in mother–child dyads and peer triads

## Comparative framework and the evolutionary trajectory

The third and final aim of this review was to link the summarized findings on turn-taking comprehension and production and involved milestones to inform studies tackling the evolution of turn-taking. To do so, we will adopt a recent comparative framework (Pika et al., 2018) that offers to enable systematic comparisons of four specific hallmarks of human turn-taking across species and taxa. By adopting such a universal framework, the field of developmental turn-taking can unify in a direction that would partially satisfy our fifth limitation, as outlined in the “Limitations and Research Bias” section above, which addressed universal coding schemes and cohesive study designs and has been considered to be vital for cross-cultural comparisons in humans (Liesenfeld & Dingemanse, 2022; Wen et al., 2025). It is important to note that the comparative framework is such—a framework and not an independent model of turn-taking. This framework is built upon traditional models of social interaction and turn-taking (Duncan, 1972; Duncan & Fiske, 1977; Duncan & Niederehe, 1974; Leclère et al., 2014; Oullier et al., 2008; Sacks et al., 1974; Tognoli et al., 2020; Verga et al., 2023). Below, we first briefly present the framework, define each individual element, and explain in more detail how they can be operationalized in developmental studies of human interaction.

The comparative framework by Pika and colleagues (2018; see also, Vlaeyen et al., 2025) emphasizes four key elements characterizing human social action during conversations based on the classical model of turn-taking by Sacks and colleagues (1974): 1) Flexibility of turn-taking organization (how is it organized), 2) participation frameworks (who is taking the next turn), 3) temporal relationships (when do response turns occur), and 4) adjacency pair-like sequences (what should the next turn do; Pika et al., 2018).

The first element, 1) flexibility of turn-taking organization, refers to underlying organizational structure, and thus the variability of the size of turns and ordering of turns. It also addresses the ability to voluntarily change and adjust signals/actions, and thus the degree of underlying cognitive flexibility and intentionality involved (Pika et al., 2018). In comparing this element to human conversational turn-taking, a number of the turn-taking requirements, as identified by Sacks and colleagues (1974), are relevant to this element including speaker change; variability of turn order, size of turn, length of interaction, content of turn, relative distribution of turns; continuity of interaction; and turn-constructional units (see Table 1 for an overview of all requirements for a model of turn-taking).

Concerning turn-taking development, a special focus for future studies thus would be to investigate whether the variety and site or ordering of turns changes during development (e.g., variety increases or is fine-tuned or ordering changes from simple to complex), how the onset of intentionality impacts upon turn-taking comprehension and production, and which role the onset of distinct cognitive skills (e.g., joint attention, theory of mind) play. In addition, these aspects may differ in relation to the used modality (i.e., verbal, gestural, or multimodal) for both the initiating and responding turn and the speaker order (either alternating or repeating speaker). Also included fillers and filled pauses, such as *uh*, *mhm*, or *um* (Skantze, 2021), may be strongly influenced by development and accompanying cognitive skill. By operationalizing this element, turn-taking interactions between infants and children and their caregivers or peers can be analyzed in both a qualitative or quantitative manner, and be systematically compared to turn-taking interactions and involved skills of other animal species.

The second element, 2) participation frameworks, is concerned not only with who is initiating the next turn, but also with the signals and techniques used to allocating turns (Pika et al., 2018). This element includes turn-taking model requirements such as turn order, distribution of turns among interactants, continuity of the interaction, turn-constructional units, as well as turn-allocation techniques and repair mechanisms. Furthermore, the presented functional concepts of repair and gaze cues, prosodic cues, verbal cues, and gesture cues are also included in this comparative element. This particular element can be very useful for future studies on turn-taking development to gain a detailed understanding of the role of distinct signal modalities, signal categories (e.g., tactile vs. visual) to initiate and allocate turn-taking, and involved methods of correcting turn-taking violations. In addition, it can be used to tackle different social factors including an interaction such as individual and social attributions of interactants involved (e.g., rank, sex, status), and their relationship (e.g., friends, professional relationship, power).

The third element, 3) temporal relationships, concerns the time window between the onset/offset of a signal or an action, and the onset of the response, which can be species specific (Pika et al., 2018), and possibly modality and context specific (Vlaeyen et al., 2025). This enables the assessment of whether interactions, turn-taking systems, specific dyads, and individuals adhere to specific temporal principles, thereby also allowing the investigation of the functional concepts of gaps and overlaps (cf. Skantze, 2021). As described above, gaps and overlaps describe how answers are given, if they are too early (overlapped), latched, contingent, or late. However, as criticized in the “Timing” section above, the distinction of what is considered contingent and what is considered late has not been clearly defined in the literature and can vary based on the modality speaker’s action and the modality of the response to that action (Keller et al., 1999; Van Egeren et al., 2001). This particular aspect has been widely investigated in both human and nonhuman turn-taking research, but has rarely been compared across species, contexts, and interactional settings (Fröhlich et al., 2016; Pika et al., 2018). The inclusion of this element also allows for comparative measures of the development of turn-taking latency across species in an organized, consistent, and bottom-up manner.

The fourth and final element, 4) adjacency pair-like sequences, concerns the structure of interaction and is based on adjacency pairs characterizing human conversations (Schegloff, 2007). These involve a minimum of two turns by different individuals, where a *first pair* part (e.g., request) is answered by an expectable response in the form of a *second pair part* (e.g., granting; Pika et al., 2018). For turn-taking, this element is particularly important to investigate what type of response is expected to follow a particular signal/action, such as question–answer, offer–acceptance/rejection, request–acceptance/rejection, greeting–greeting response, inform–acknowledge, and call–beckon pairings to name a few (Levinson, 2013). Concerning developmental studies on turn-taking, this element can be used to examine whether the production, usage, and comprehension of adjacency pairs is linked to development, cognitive skills, and whether the variety is linked to age, sex, context, and specific social attributes of interactants or the involved dyad.

In sum, the comparative framework offers a useful toolbox to tackle the development of turn-taking and the role of accompanying cognitive skills and social attributes, enabling the drawing of systematic quantitative and qualitative comparisons between and within species. It thus represents a unique opportunity to expand the understanding of not only human ontological conversational turn-taking but also to tackle the evolutionary trajectory of turn-taking. In adopting and operationalizing key elements of the comparative framework, it may be easier for researchers to consider more holistic perspectives and interpretations of turn-taking in both production and comprehension even when the research focus is on a single functional concept or cue. Furthermore, a universal, bottom-up approach to the coding of turn-taking would unify observational approaches to the investigation of turn-taking, without redefining turn-taking or taking away from the importance of functional concepts and cues of turn-taking (Skantze, 2021) or existing models of turn-taking (e.g., Sacks et al., 1974). These implications not only aid in the verification of the milestones presented in the “Milestones of Turn-Taking in Development” section, but they also would allow for cross-cultural corpora to be cultivated, further allowing for meta-analyses to be conducted (Liesenfeld & Dingemanse, 2022) and to naturally consider multimodal coding schemes (cf. Gabouer & Bortfeld, 2021; Levinson, 2024; Rohlfing et al., 2020). By adopting the simplistic comparative framework, we can not only bridge the gap between disciplines, but also further broaden our scope, which could lead to a gain in the comprehensive understanding of the evolutionary trajectory and even possible precursors of human conversational turn-taking as well as attain a more holistic perspective across research endeavors.

## Discussion

In this review, we had three specific aims: First, to review the existing literature on turn-taking in development from 0–6 years of age and pinpoint limitations and research biases. Second, to extrapolate potential developmental milestones in the comprehension and production of turn-taking from the existing literature. Third, to link the presented studies to a recent comparative framework enabling a new tool for direct, systematic comparisons between species and taxa and thus an informed understanding of the phylogenetic history of turn-taking. In the following sections, we will discuss each of these aims in turn, present limitations of the current review, and provide considerations and recommendations for future research.

The first aim of this review was to review the developmental literature of turn-taking. To this end, we divided the research into two separate sections: comprehension and production. In our review, we found that even as neonates, infants are able to comprehend and engage in alternating interaction in a structured manner (Dominguez et al., 2016; Guellai & Streri, 2011; Nagy, 2006; Nagy & Molnar, 2004). We have also found that while there is a vast range of literature in the development of turn-taking in terms of functional concepts and cues (cf. Skantze, 2021), there is simultaneously little research. In the literature review, we found that many aspects of turn-taking were not equally investigated both within comprehension or production as well as across comprehension or production. In the comprehension of turn-taking, we see that timing, gaze cues, and verbal cues represent the majority of research, while the comprehension of repair mechanisms in turn-taking has not been examined in children up to the age of 6 years. In the production of turn-taking, we found a broader spectrum of research in the developmental literature as it pertains to all functional concepts and cues to turn-taking. However, there are underrepresented cues in the production of turn-taking—namely, the production of prosodic cues specifically in alternating social interactions. Furthermore, we also found that, like repair mechanisms, gestural comprehension and production in turn-taking is lacking in the developmental literature. As such, we have identified gaps in the literature that can guide future research.

In addition to gaps in the literature, we have also found that many influential factors of turn-taking in development (e.g., SES, IDS, linguistic development, social development, the development of dynamic models linked to turn-taking) have not been broadly considered and represented in the research. To this end, there is also a broad variation in methodological approaches. These various approaches may be valid, but it remains difficult to compare and contrast findings when methodologies and operationalizations vastly differ. This was also seen in the consideration in timing as to what are the limitations of a contingent response. To circumvent these differences and allow for comparisons between methodologies to be made, meta-analyses are a great tool. However, there are few meta-analyses in the development of turn-taking. Furthermore, the low number of longitudinal studies makes it difficult to map out a trajectory of turn-taking development, particularly on an individual scale. We hope by addressing these gaps in the literature creates an opportunity to fill them and to inspire researchers to address these issues in future research.

The second aim of this review was to extrapolate possible developmental milestones in both comprehension and production of turn-taking in both functional concepts as well as cues to turn-taking. In order to meet this goal, we identified the first evidence of comprehension or production of a specific aspect of turn-taking in the literature; however, the further development of these aspects is not defined in the milestones. Although we were able to identify quite a number of milestones in the literature, these remain hypothetical and need to be subjected to further investigation by means of direct testing, by means of meta-analysis, observational studies, and experimental exploration. The authors hope that the initial indication of milestones will help guide research in the field of developmental turn-taking in order to create a more complete understanding of how turn-taking is learned and produced from birth to maturation of the skillset.

The third and final aim of this review was to introduce the comparative framework of Pika and colleagues (2018) to the field of developmental turn-taking in humans, offering a new tool box to enable informed, systematic within and cross-species comparisons. The results will equip us with crucial insights into evolutionary precursors of turn-taking in closely related and other animal species, and offer the possibility to verify whether cooperative turn-taking indeed represents an ancient layer of the language system (Levinson, 2016). In addition, the application of the comparative framework, and its expansion to humans, will also allow for a more systematic, holistic assessment of turn-taking systems and involved elements and cognitive skills across human cultures and languages, thereby allowing for a systematic quantitative assessment of the ontogenetic trajectory of turn-taking and its importance for the evolution of language.

We were able to review the literature on developmental turn-taking, achieving our three goals; however, our review is limited in its interpretation of turn-taking, identification of developmental milestones, and the application of a comparative framework. In the review of turn-taking, we were only able to describe the features, function, and acquisition of turn-taking as it was described in the literature. However, in the study of developmental turn-taking, there are many scientific gaps in the literature where research and understanding has yet to be explored. As such, a complete picture of the development of turn-taking cannot yet fully form. Our review is a step to identify, and ultimately help to fill, these gaps. In the identification of the developmental milestones of turn-taking, we were limited to the current literature and how turn-taking was studied and the age groups selected within those studies. Thus, we are limited in our declaration of the milestones, as we rely on the current state of the literature. Furthermore, we recognize that the milestones are currently subjective to the current literature review and there is a need for experimental validation of the milestones identified. Moreover, there are both comprehensive and productive milestones that are missing, which need to be addressed in future research. In the justification for a comparative framework (Pika et al., 2018), we are also limited in this review, as we did not experimentally apply the framework to human turn-taking. This should be done in an experimental setting for both the comprehension and production of developmental turn-taking, ideally in a longitudinal experimental or observational design.

In compiling the current review, we additionally identified five limitations of the current body of literature, which together with our three main goals form considerations and concrete recommendations for future research. The literature limitations we identified were 1) a clear lack of longitudinal studies, 2) gestures and multimodal signals being overlooked, 3) a lack of interactions between functional concepts and cues of turn-taking in development, 4) a need for cultural specific research and cross-cultural comparisons, and 5) a need of a culturally sensitive and standardized coding scheme and cohesive experimental designs. Based on these limitations and the gaps in the literature identified in the review, we hope to have stirred more interest with researchers considering aspects such as sample size, age groups, culture, methodology, statistical analyses, and base assumptions. As outlined in the “Milestones of Turn-Taking in Development” section, these factors can play a role in research and in the identification of developmental milestones, yet the extent of their influence is not fully defined in the current review. For this, a methodological meta-analysis should be conducted. Furthermore, we have clear recommendations for future research:

1. We recommend the conduction of longitudinal studies for all feature and cue development in turn-taking. By doing so, the field can gain insight into the individual development of turn-taking as well as verify, challenge, expand the developmental milestones suggested by this review. This would satisfy limitation one.
2. Due to the scarcity of information on gestural cues and multimodal cues in turn-taking, we recommend the inclusion of multimodal cues into turn-taking research (cf. Levinson, 2024; Rohlfing et al., 2020). This would satisfy limitation two.
3. In order to further test the validity of our suggested milestones and identification of additional milestones, we recommend that meta-analyses be conducted. Wherein we further recommend the creation of intercultural corpora (cf. Dingemanse & Liesenfeld, 2022; Liesenfeld & Dingemanse, 2022). An additional methodological meta-analysis would show the distribution of how each of the comprehensive and productive functional concepts and cues to turn-taking were attained, being better able to provide methodological recommendations as outlined above. With the creation of the corpora, we also recommend that interactions between functional concepts and cues of turn-taking and their development be investigated. This would satisfy limitation three. Furthermore, both the corpora and the information extracted from the corpora could be vital for real-world applications, such as improving human–robot interactions with children, specifically in creating more naturalistic conversation structures (cf. Baxter et al., 2013; Murali et al., 2023; Skantze, 2017, 2021).
4. We recommend expanding research not only to other cultures, but specifically to non-WEIRD cultures in an appropriate manner (for specific guidelines, see Wen et al., 2025). The ultimate goal is to be able to compare culture- or language-specific data with other datasets to find language/culture specific turn-taking functional concepts and distribution/realization of cues as well as universal functional concepts and the distribution/realization of cues. This would satisfy limitation four.
5. It is our recommendation that a universal, culturally sensitive, and multimodal coding scheme be developed for turn-taking (cf. Gabouer & Bortfeld, 2021; Gabouer et al., 2020; Liesenfeld & Dingemanse, 2022; Wen et al., 2025). One step in this direction is the application of the comparative framework by Pika and colleagues (2018), which offers a simple, bottom-up, ecological framework built on existing models of turn-taking. By applying the comparative framework as a basis of a universal coding scheme, the field could not only investigate the ontogenetic development of turn-taking, but also the phylogenetic development of turn-taking by means of cross-species investigations and comparisons. This refers to limitation two and four and would satisfy limitation five.

For future research, this review serves as a collection of current knowledge on the development of turn-taking, serves as a means of identification of scientific gaps in the literature and shortcomings of the current breadth of our understanding of developmental turn-taking, and provides concrete recommendations for future investigations. In our review and identification of developmental milestones, we found that not only are there gaps in the literature, but there is also a need for systematic longitudinal studies in the field. We have presented much information on developmental turn-taking, but an overview of how these aspects of turn-taking develop over time within a single population and in a specific social and linguistic community is lacking and necessary. Furthermore, aspects of turn-taking have been studied in observation and experimentally manipulated; yet, there is a need for a multimodal understanding of turn-taking in how these aspects interact with one another (Levinson, 2024; Rohlfing et al., 2020). Additionally, research in the development of turn-taking could benefit from a holistic approach to the statistical analysis of turn-taking and would shed light into the interaction between age, linguistic development, and between functional concepts of and cues to turn-taking. This review should guide future research in filling these scientific and statistical gaps in the literature.

## Data Availability

Not applicable as no data was used in this review paper.

## Code availability

Not applicable as no codes were used for this review paper.
